# The temporomandibular joint in juvenile idiopathic arthritis: frequently used and frequently arthritic

**DOI:** 10.1186/1546-0096-7-11

**Published:** 2009-05-29

**Authors:** Sarah Ringold, Randy Q Cron

**Affiliations:** 1 Department of Pediatrics, Division of Rheumatology, Seattle Children's Hospital, Seattle WA, USA; 2 Department of Pediatrics, Division of Rheumatology, University of Alabama at Birmingham, Birmingham AL, USA

## Abstract

Recent recognition of the markedly high prevalence of temporomandibular joint (TMJ) arthritis in children with juvenile idiopathic arthritis (JIA) coupled with the significant morbidity associated with TMJ damage has prompted increased interest in both the clinical and pathological aspects of TMJ arthritis. This review focuses on the prevalence of TMJ arthritis in JIA, the imaging modalities used to detect TMJ arthritis, and the treatment of TMJ arthritis in children with JIA.

## Background

TMJ arthritis in children with chronic arthritis was first reported by Still in his initial case series in 1897 [1]. Although TMJ arthritis in JIA is frequently asymptomatic, the TMJ is particularly susceptible to damage from arthritis due to its unique anatomy and biochemical composition. Unlike other synovial joints, the mandibular growth plate lies under a thin layer of fibrocartilage located at the surface of the condylar head [2]. Mandibular growth occurs within this center from the prenatal period until just after puberty, and damage to the growth center due to inflammation or trauma during this time period frequently results in alterations in mandibular growth [3]. In JIA, this damage has been associated with a number of clinically significant outcomes, including decreased chewing ability, malocclusion, and micrognathia. These outcomes are not infrequent, and prior studies have reported micrognathia in approximately 30% of children with JIA and malocclusion in approximately 66% [4,5]. Furthermore, even apparently minor radiographic changes have been associated with disrupted mandibular growth and a number of significant craniofacial changes [6].

Children with JIA also have a higher prevalence of headache, neck pain, and jaw dysfunction than healthy controls, both at the time of diagnosis of their TMJ disease and at long-term follow-up [7,8]. A recent prospective cohort study of children with JIA and healthy controls which reported data on patients and controls at baseline and after 15 years of follow-up found that, although there were no difference in TMJ-related symptoms at baseline, after 15 years children with JIA reported a statistically significant higher prevalence of tiredness of the jaw (46%, p = 0.04), pain in face or jaw (39%; p = 0.02), and difficulty in opening their mouth wide (36%; p = 0.02). In addition, these children had an increased prevalence of additional symptoms that may be associated with TMJ disease, including tooth clenching, headache, neck pain, shoulder pain, and impaired general health at follow-up [7]. These findings, coupled with the fact that the TMJ is one the most frequently used synovial joints in the body, used up to 2,000 times per day for chewing and speaking, highlight the importance of recognizing and treating TMJ arthritis during childhood.

### Prevalence and incidence of radiographic imaging abnormalities, symptoms, and physical exam findings

Initial estimates of the prevalence of TMJ involvement in JIA varied widely, ranging from 25–70%, and were based on cross-sectional analyses primarily using orthopantomograms (OPTs) for diagnosis [4,9,10]. The large variation in estimates likely resulted from differences in inclusion criteria, including variable types of JIA, and differences in disease duration (Table 1). Measurement of the prevalence of TMJ disease has been further complicated by the observation that TMJ symptoms and physical examination are not reliable for the assessment of TMJ arthritis and a large proportion of children may be asymptomatic and/or have a normal TMJ examination despite radiographic evidence of TMJ damage. In a prior series, as many as 69% of children with evidence of TMJ damage on OPT have been found to be asymptomatic [11,12]. Although longer disease duration, younger age at disease onset, decreased mouth opening, decreased or absent translation, pain with mandibular excursion, and crepitus have all been associated with the presence of TMJ damage, the heterogeneity of prior studies makes interpretation of these results difficult [10,12,13]. Given the high prevalence of TMJ involvement in JIA, and because many children are asymptomatic despite TMJ damage, investigations have sought to identify specific patient and laboratory characteristics associated with the development of TMJ arthritis. Such patient and laboratory characteristics may help to identify patients at highest risk of TMJ involvement and those most likely to benefit from screening or early intervention.

**Table 1:** Prevalence of temporomandibular joint (TMJ) radiographic damage, symptoms, and physical exam findings in children with juvenile idiopathic arthritis (JIA): results from selected studies.

Reference	Study Sample	Inclusion Criteria* & Study Design	JIA Type^‡^	Imaging Modality	TMJ Damage**	TMJ Symptoms	Abnormal TMJ Exam
[9]	*N*: 249 *Female*: 66%	- Suspected TMJ involvement - Cross-sectional	NA	OPT	Overall 28.9% Female 75%	10.8%	< 40%

[12]	*N*: 20 *Female*: 95% *Mean age*: 24.5 years *Mean age JIA onset*: 5.3 years	- Micrognathia - Cross-sectional	Multiple	OPT	75% (defined as complete resorption of the condylar head)	65% (at anytime during JIA course)	55%

[16]	*N*: 15 *Mean age*: 12 years	- JIA duration ≤ 3 years; Age ≥ 8 years - Prospective cohort	NA	OPT; MRI with gadolinium	87% (MRI) 40% (OPT)	3.5%	60%

[4]	*N*: 169 *Female*: 67% female *Mean age*: 9.8 years *Mean age disease onset*: 5.4 years	- Consecutive referrals to an orthodontic clinic - Cross-sectional	Multiple	OPT	Female 73.3% Polyarticular 65.8% Oligoarticular 44.5% Systemic 42.9% ANA+ 55.1% HLA-B27+ 38.5%	NA	NA

[10]	*N*: 97 *Female*: 62%	- Routine referrals to an orthodontic clinic - Cross-sectional	Multiple	OPT	47% Oligoarticular 39% RF-polyarticular 59% RF+ polyarticlar 33% Systemic 67% Enthesitis related arthritis 13% Psoriatic 33% Other 50%	9–18%	16–39%

[6]	*N*: 46 *Female*: 63% *Median age*: 9.33 years	- Consecutive children consented to OPT - Cross-sectional	Multiple	OPT	78%	70% (signs symptoms reported together)	NA

[15]	*N*: 66; *Female*: 59.1% *Mean age*: 11.9 years	- Inclusion criteria NA - Cross-sectional	Oligoarticular Polyarticular	OPT	Polyarticular 75%, Oligoarticular 20%	NA	NA

[17]	*N*: 46 *Female*: 59% *Median age*: 12.7 years *Median age JIA onset*: 4.5 years	- Inclusion criteria NA - Cross-sectional	Oligoarticular Polyarticular Systemic (polyarticular course)	MRI with gadolinium	32% (abnormal condyle) 45% (pannus)	5.5–8.3%	9% (decreased mouth opening)

[18]	*N*: 32 *Female*: 78% female *Mean age*: 8.6 years	- Newly diagnosed JIA - Cross-sectional	Multiple	MRI with gadolinium	75% (effusion or synovial thickening) 69% (condylar changes) 53% (both)	19%	41%

JIA: Juvenile idiopathic arthritis, OPT: Orthopantomogram, MRI: Magnetic resonance imaging, NA: Not available.

*All studies required diagnosis of juvenile arthritis for inclusion.

^‡^Multiple indicates > 3 JIA types represented.

**Percentages are based on the number of children with the specified characteristic.

Data from recent reports have confirmed the relatively high prevalence of radiographic evidence of TMJ involvement in JIA and the low prevalence of patient-reported TMJ symptoms and/or physical exam abnormalities. A recent cross-sectional report of 100 children with multiple JIA types and different disease durations reported condylar damage on the OPTs of 78% of the 46 children who had OPTs performed [6]. In this cohort, 78% of the children who had OPTs performed had evidence of bilateral TMJ involvement. Furthermore, although a relatively large proportion of the entire cohort (70%) reported TMJ symptoms, there was no association between patient-reported symptoms and the presence of damage on OPT. There was also no association found between disease activity (dichotomized as active disease versus inactive disease based on the criteria published by Wallace and colleagues [14]), disease duration, or JIA subtype and the presence of unilateral or bilateral damage. In a similar series of 66 children with polyarticular or oligoarticular JIA, condylar damage on OPT was reported in 50% of the cohort and 35% of this group had bilateral disease [15]. Seventy-five percent of the children with polyarticular JIA had evidence of TMJ damage and 20% of the children with oligoarticular JIA had damage. There was no association between RF, ANA, or HLA-B27 positivity and the presence of TMJ damage. While girls in this cohort tended to have a higher prevalence of TMJ damage, this finding was not statistically significant. Children with polyarticular JIA and children with longer disease duration in this cohort were more likely to have bilateral disease. In contrast, Pederson and colleagues reported that, in a cohort of children with JIA referred for orthodontic evaluation, children with longer disease duration and polyarticular course were more likely to have extensive damage on OPT, while those children who were ANA positive or HLA-B27 positive tended to have less TMJ damage visible on OPT [4]. Although these OPT-based studies reflect a degree of selection bias, they also likely under-represent the extent of TMJ arthritis, as OPT detects evidence of arthritis only after bone damage has already occurred [8].

Studies using magnetic resonance imaging (MRI) with gadolinium enhancement have provided even higher prevalence estimates. A prospective cohort study of 15 children with JIA followed with MRI over 2 years reported that, although the MRI findings tended to fluctuate over time, 14 of the children had evidence of synovial enhancement during the study period [16]. Twenty-five percent of the children in this cohort had normal TMJ exams throughout the follow-up period and 70% of children were asymptomatic. Detailed TMJ exams were performed by the same orthodontist at 6–8 weeks and included assessment of tenderness, swelling, loss of range of motion, and asymmetry. In a more recent, cross-sectional report of 46 children with JIA and widely varying ages (2–37 years) and disease durations (0.3–24.8 years), 32% of participants were reported to have abnormal condyles, 10% had intra-articular fluid, and 45% had enhancing pannus [17]. In this cohort, systemic JIA with a polyarticular course, younger age at onset, and longer disease duration were independent predictors of condylar damage. In a separate report, Weiss and colleagues prospectively screened a cohort of 32 children with newly diagnosed JIA by MRI with gadolinium within 8 weeks of their diagnosis. Seventy-five percent of this cohort had evidence of active TMJ arthritis, defined as effusion and/or synovial thickening, and 69% of this cohort had evidence of chronic TMJ arthritis, defined as abnormal condyles or condylar erosions [18]. Despite the high prevalence of TMJ disease in this cohort, 81% of the children had no TMJ symptoms and 59% had a normal TMJ examination performed by a pediatric rheumatologist, which included assessment of mouth opening, pain, asymmetry, translation, and micrognathia. The positive predictive value (PPV) for TMJ symptoms was 100% whereas the negative predictive value was only 32%. Similarly, the PPV of a normal TMJ exam was 69% and the negative predictive value was only 21%. Eighty-one percent of children with acute TMJ arthritis had bilateral involvement and 68% of children with evidence of chronic TMJ arthritis had bilateral disease. These results were supported by a recent report by Muller and colleagues in which 30 consecutive JIA patients were evaluated by physical exam, orthodontic exam, TMJ ultrasound (US), and TMJ MRI [19]. Sixty-three percent of children in this cohort were determined to have active TMJ disease by MRI. Using MRI as the gold-standard for detection of TMJ disease, the authors reported rheumatological exam had a PPV of 0.85 (0.54–0.97) for the detection of active TMJ disease, orthodontic exam had a PPV of 0.56 (0.31–0.79), and US had a PPV of 0.75 (0.36–0.96). The highest predictive value was for abnormal maximal incisal opening (MIO), and an MIO of < 40 mm had a PPV of 1 (0.56–1.0).

Given the challenges of identifying children with TMJ involvement and those at highest risk of developing TMJ disease, it has been difficult to estimate the incidence of TMJ disease in JIA and very few reports have specifically addressed this question. Recently, Twilt and colleagues screened a series of 89 JIA patients with yearly OPTs and reported the incidence of condylar damage to be 0.071 per patient year in the subgroup of 48 patients who had no evidence of TMJ damage by OPT at baseline [20]. However, screening by MRI with contrast or ultrasound, as discussed below, may yield notably higher incidence rates of TMJ disease in children with JIA than those generated by screening by OPT.

### Radiographic imaging

Because the signs and symptoms of TMJ arthritis have been found to be unreliable, radiographic imaging plays a central role in the diagnosis and follow-up of TMJ arthritis. OPT and computed tomography (CT) scanning are both useful in delineating the extent of condylar damage. Computed tomography is generally preferred to OPT because of the shorter exam time and lower radiation dose. However, these modalities cannot distinguish damage due to past disease activity from that associated with ongoing, active disease. Nor can these modalities detect early changes, such as synovial inflammation. To date, scoring systems for condylar damage have been proposed for US, CT, and MRI in TMJ disease [21-23]. Conversely, although it's detection of condylar damage is limited, MRI performed with gadolinium and ultrasound (US) can detect joint effusion and pannus formation, and therefore can be used to determine whether the arthritis is active [24]. The primary disadvantages to MRI are that younger children may require sedation for the exam and cost is higher than other imaging modalities. Moreover, optimal MRI exams require MRI machines equipped with TMJ-specific surface coils.

Several prior reports have directly compared US and MRI and have reported varying sensitivities and specificities, likely reflecting the operator-dependent nature of US as the high sensitivities for US were reported in studies from Europe where there is more experience with this modality [25,26]. The use of US in this case is also limited by the anatomy of the TMJ, which allows for probe placement only on the most lateral aspect of the joint. Most recently, US and MRI were directly compared in the series of 32 children with newly diagnosed JIA (described above) [18]. While 75% of children had evidence of active disease by MRI, no children were diagnosed with active TMJ arthritis by US (kappa = 0). Likewise, 69% of participants were found to have evidence of chronic TMJ arthritis on MRI, but only 28% were diagnosed by US (kappa = 0.12). Thus, for most centers, MRI seems to be far more sensitive than US at detecting TMJ arthritis in children with JIA.

Additional studies have attempted to correlate particular physical exam findings and disease characteristics with specific radiographic abnormalities. The series by Billiau and colleagues described above reported that decreased MIOs were more common in children with active disease [6]. One study of 15 children with JIA and minimum disease duration of 3 years followed with MRI and/or OPT for 2 years reported that decreased translation (lateral movement of the mandible at maximal opening) was associated with condylar resorption visible on both OPT and MRI [8]. Furthermore, in this small study, decreased mouth opening was significantly associated with condylar resorption on both MRI and OPT at baseline, but only with OPT at the end of the study. Interestingly, overall TMJ function improved in this cohort over the 2 year follow-up. Mouth opening in this cohort was similar to healthy controls by the end of the 2 years of follow-up, even though the majority of children (80%) had condylar changes on MRI by the end of the study. Based on these observations, the authors proposed the use of translation and mouth opening as a screening technique for early TMJ disease. Argyropoulou and colleagues also reported a significant association between decreased mouth opening and condylar damage on MRI in their series of patients described above [17]. One study of 48 children screened by US reported that children with ≥ 5 active joints were more likely to have US evidence of TMJ arthritis, children with disease duration more than 23 months were more likely to have disc dislocation and/or condylar damage, and children with disease duration more than 60 months were most likely to have condylar damage [27]. Moreover, since three-quarters of the children with JIA were found to have active TMJ disease at onset by MRI, the vast majority of which were asymptomatic, it could be argued that perhaps all children with newly diagnosed JIA should be screened by this approach [18]. In the few patients with follow-up MRIs post-IAS treatment, improvement in the TMJ arthritis was frequently noted [18].

There are to date few descriptions of the evolution of the radiographic findings over time. Twilt and colleagues reported improvement in 27 out of the 89 patients who were followed up for a mean of 14 months [20]. Nineteen patients had normal OPTs at the end of the study and 4 patients had evidence of worsening changes. Disease activity, as measured by physician-reported visual analog scale, was lower in the patients who demonstrated improvement. Systemic medication use in this cohort was not reported; however, a substantial number of patients had splinting and none of these patients underwent IAS during the study period. After 5 years of follow-up of this same cohort, 83% of patients had evidence of improvement of their condyles on OPT, and the overall prevalence of abnormal condyles in the study cohort decreased from 49 to 40%, again indicating a trend towards improvement in previously visualized damage [28]. Although children with TMJ damage were more likely to receive immunosuppressive medications and biological agents, treatment effects could not be assessed in this study. Moreover, the response of TMJ arthritis to systemic TNF inhibitory therapy in children with JIA is unknown at present.

Recently, cone-beam CT has been proposed as a novel way of delineating the extent of condylar damage. This technique uses specialized CT to create detailed images of the condyle and estimates of condylar volume and results in lower radiation exposure to the patient than conventional CT [29]. Twenty children were evaluated with this technique. JIA subtype and disease history were not detailed for the patients. The investigators reported significant asymmetry, defined as differences in shape or volume, of the condyles of these children. At present, this degree of detail may be more useful for research rather than for routine clinical care [30].

### Treatment

Historically, TMJ arthritis in children has been treated with splinting and/or surgical techniques intended to compensate for the poor mandibular growth, with variable outcomes [31].

#### Systemic therapies

Systemic medications have not been extensively evaluated for TMJ arthritis. One non-randomized series of children with polyarticular or oligoarticular JIA and TMJ involvement reported weekly methotrexate may result in decreased TMJ destruction and decreased craniofacial alterations [32]. Combination therapy with methotrexate and infliximab has shown promise for the treatment of TMJ disease in a small pilot study in adult RA, but has not been specifically evaluated in children [33]. Moreover, because of the anecdotal experience of children developing TMJ arthritis while on TNF inhibitors, more directed therapy for the TMJ may be required.

#### Intraarticular therapies

Although IAS have been shown to be safe and effective for peripheral joint arthritis in JIA, there has been some concern about their use in TMJ arthritis due to reports suggesting potential risks in non-inflammatory TMJ conditions [34,35]. One case report described the development of ankylosis in an adult with TMJ dysfunction secondary to trauma treated with 15 IAS over a two year period [36]. Another case series of adults with TMJ dysfunction due to presumed osteoarthritis treated with IAS reported damage to the fibrous layer of the condylar head and the cartilaginous zones of the injected joints [37]. However, a subsequent series that evaluated the effects of TMJ IAS in adults with TMJ arthritis reported an improvement in patient symptoms and bite force in patients who completed the two year follow-up [38]. A more recent case report of a child with TMJ arthritis treated with IAS and synovectomy reporting sustained clinical and radiographic improvement following these procedures, and the continued success of IAS in the treatment of peripheral joints in JIA has encouraged further exploration of this treatment modality [39].

**Figure 1:**
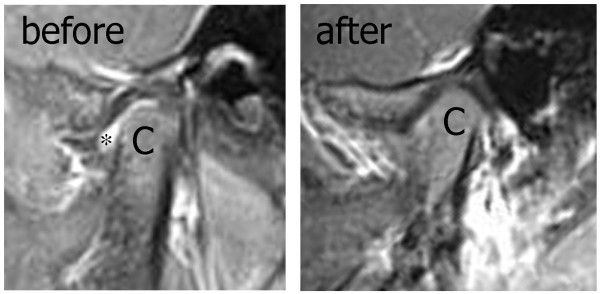
**Post-contrast, fat-saturated MRI images of an arthritic TMJ in a child with JIA before (left) and after (right) intra-articular corticosteroid injection**. The head of the condyle (C) and intra-articular fluid (*) prior to the injection are noted.

Recently, the safety and efficacy of TMJ IAS in JIA was evaluated in a group of 23 children with JIA and evidence of effusion or pannus formation on contrast-enhanced TMJ MRI [40-42] (Table 2). These children were treated with either triamcinolone acetate or triamcinolone hexacetonide IAS performed with CT guidance. Following IAS, 10 children had an increase in mouth opening of 5 mm or more, with children less than 6 years of age having the greatest increase in mouth opening. Ten of the 13 children who had symptoms at the time of IAS reported improvement following the procedure. Eleven of the 14 children who had follow-up MRIs obtained after the procedure had resolution of their joint effusions (Figure 1). A retrospective case series of 25 children with multiple subtypes treated with one or more TMJ IAS performed without radiographic imaging reported a similar increase in mouth opening following IAS (mean 3.8 mm) [43] (Table 2). Increase in mouth opening was greatest after first IAS (mean increase 6.6 mm) but increase after subsequent injections was only 0.4 mm, despite the majority of mouth opening measurements being low for age prior to these subsequent injections. Although follow-up imaging was not routinely obtained for this cohort, two patients had improvement in their CT imaging findings, 3 patients had stable damage, and 10 patients had worsening TMJ damage. Adverse events were rare in both series and included transient facial swelling in 2 patients and subcutaneous atrophy at the injection site in one patient who underwent 5 IAS. Data from 2 additional series have been recently presented in poster format and also support these findings (Table 2). Although these results suggest that IAS may have utility in treating TMJ arthritis in JIA, the studies were not able to control for systemic medication use or non-treatment related changes that may occur in the TMJ over time. Ultimately, comparison of systemic therapies with or without IAS for TMJ arthritis in JIA, along with longer-term follow-up, is still required.

**Table 2:** Summary of series describing the outcomes of intra-articular corticosteroid injections of the temporomandibular joint (TMJ) in JIA

Series	No. of patients/No. of injections	Diagnostic Imaging Modality	Duration of Follow-up	Selected Outcomes	Adverse Events	Intra-articular injections
[41]	21/36	MRI with gadolinium	Follow-up clinical assessment: Median: 42 days Range: 1–164 days Follow-up imaging: Mean: 52 days Range: 21–125	1. Pain resolved in 5 patients. 2. Tenderness resolved in 7 of 11 patients. 3. Mean MIO increase of 1.8 mm (p = 0.16 compared to controls) 4. MRI improvement in 23 of 36 joints. 5. Synovial enhancement resolved in 6 of 36 joints.	NA	Injections with triamcinolone hexacetonide.

[43]	25/74	CT	Mean: 26 mos Range: 5–52 mos	1. 21 of 25 patients asymptomatic at end of study period (10 of 25 normal prior to injection). 2. 18 of 25 patients with normal TMJ exam at end of study period (10 of 25 normal prior to injection). 3. Mean MIO increase of 6.6 mm after first injection. 4. Mean MIO increase of 0.4 mm after subsequent injections	1 patient with subcutaneous atrophy at injection site (after 5 injections) 2 patients with asymptomatic calcifications	Injections with triamcinolone acetonide or triamcinolone hexacetonide. Performed without radiographic guidance.

[42]	10/16	MRI with gadolinium	3 mos	1. Synovial enhancement resolved in 16 of 16 joints. 2. Improvement in asymmetric mouth opening in 3 of 4 patients.	None	Injections with triamcinolone acetonide.

[40]	23/40	MRI with gadolinium	6–12 mos	1. Pain resolved in 10 of 13 patients. 2. Mean MIO increase of 5 mm. 3. Resolution of effusion in 11 of 23 joints.	2 patients with short-term facial swelling	Injections with triamcinolone acetonide or triamcinolone hexacetonide. Performed with CT guidance.

MRI: Magnetic resonance imaging; MIO: Maximal incisal opening; CT: Computed tomography

#### Treatment data from animal models

In contrast to these studies in children with JIA, researchers in Denmark have studied the efficacy of TMJ IAS in a rabbit model of antigen induced TMJ arthritis. One report compared control rabbits to rabbits with induced TMJ arthritis that were untreated, treated with saline injections, or treated with IAS [44]. The rabbits received 4 IAS or other saline injections at an interval of 3 weeks, with arthritis induced prior to each treatment. At the end of the study, rabbits that received the corticosteroid injections had decreased mandibular growth and increased posterior rotation of the mandible as compared to the 3 other treatment arms. In a second report from the same group, rabbits were treated with saline injections, ovalbumin alone, or ovalbumin and corticosteroid, and they received 4 IAS over a period of 10 weeks, with arthritis induced prior to each treatment. Rabbits treated with corticosteroids had a decreased number of plasma cells in the synovial connective tissue of the TMJ when the synovium was directly examined by an experienced pathologist, but plasma cell counts, as measured by semi-quantitative measures, and synovial thickness did not differ between treatment groups [45]. While these experiments raise questions about the efficacy of multiple IAS on the TMJ it is unclear how applicable the results from this particular induced rabbit model are to humans and to the inflammatory processes underlying JIA.

A series of 2 recent reports from this same group also compared the effects of intra-articular saline injections, intra-articular etanercept injections (0.1 mg/kg), or systemic etanercept (0.8 mg/kg) in this same rabbit model of TMJ arthritis [46,47]. Although all animals showed a similar degree of synovial proliferation and thickened synovium, the TMJs of animals treated with systemic etanercept showed a decrease in plasma cell count and inflammation, as measured semi-quantitatively, along with near normal mandibular growth parameters, as compared to those treated with intra-articular saline or etanercept. Because there was no corticosteroid treated group included, it is not possible to determine how the effects of etanercept on the TMJ differ from corticosteroid. Whether or not this particular animal model serves as a good surrogate for TMJ arthritis in children with JIA is still in question.

## Conclusion

Active TMJ arthritis is seen in as many as 75% of children with JIA, indicating that it is one of the most frequently involved synovial joint(s) in JIA. Moreover, TMJ arthritis has been described in association with all of the JIA types. MRI with contrast appears to be the most sensitive tool for detecting TMJ arthritis in JIA, and while several factors have been associated with an increased risk of TMJ arthritis, including longer disease duration, young age at disease onset, and polyarticular or systemic course, more systematic investigation into the factors associated with TMJ damage is still required. Furthermore, although the majority of children are asymptomatic at the time their TMJ arthritis is initially identified, these children are at risk of unfavorable long-term outcomes from the associated joint damage. Intra-articular corticosteroid injections have shown promise for the treatment of active TMJ disease, but additional studies of the long-term effects of IAS compared to the effects of systemic therapies will be important in determining optimal treatment of TMJ arthritis in children with JIA.

## Abbreviations

CT: computed tomography; IAS: intra-articular steroids; JIA: juvenile idiopathic arthritis; MIO: maximal incisal opening; MRI: magnetic resonance imaging; OPT: orthopantomogram; TMJ: temporomandibular joint; US: ultrasound.

## Competing interests

The authors declare that they have no competing interests.

## Authors' contributions

Both authors contributed to the collection of data and references, drafting and revising this manuscript. Both have read and approve the final manuscript.

## References

[B1] Still GF (1897). On a form of chronic joint disease in children. Med Chir Trans.

[B2] Sarnat BG (1951). The Temporomandibular Joint.

[B3] Ronchezel MV, Hilario MO, Goldenberg J, Lederman HM, Faltin K, de Azevedo MF, Naspitz CK (1995). Temporomandibular joint and mandibular growth alterations in patients with juvenile rheumatoid arthritis. J Rheumatol.

[B4] Pedersen TK, Jensen JJ, Melsen B, Herlin T (2001). Resorption of the temporomandibular condylar bone according to subtypes of juvenile chronic arthritis. J Rheumatol.

[B5] Karhulahti T, Ylijoki H, Ronning O (1993). Mandibular condyle lesions related to age at onset and subtypes of juvenile rheumatoid arthritis in 15-year-old children. Scand J Dent Res.

[B6] Billiau AD, Hu Y, Verdonck A, Carels C, Wouters C (2007). Temporomandibular joint arthritis in juvenile idiopathic arthritis: prevalence, clinical and radiological signs, and relation to dentofacial morphology. J Rheumatol.

[B7] Engstrom AL, Wanman A, Johansson A, Keshishian P, Forsberg M (2007). Juvenile arthritis and development of symptoms of temporomandibular disorders: a 15-year prospective cohort study. J Orofac Pain.

[B8] Pedersen TK, Kuseler A, Gelineck J, Herlin T (2008). A prospective study of magnetic resonance and radiographic imaging in relation to symptoms and clinical findings of the temporomandibular joint in children with juvenile idiopathic arthritis. J Rheumatol.

[B9] Ronning O, Valiaho ML, Laaksonen AL (1974). The involvement of the temporomandibular joint in juvenile rheumatoid arthritis. Scand J Rheumatol.

[B10] Twilt M, Mobers SM, Arends LR, ten Cate R, van Suijlekom-Smit L (2004). Temporomandibular involvement in juvenile idiopathic arthritis. J Rheumatol.

[B11] Larheim TA, Hoyeraal HM, Stabrun AE, Haanaes HR (1982). The temporomandibular joint in juvenile rheumatoid arthritis. Radiographic changes related to clinical and laboratory parameters in 100 children. Scand J Rheumatol.

[B12] Larheim TA, Haanaes HR (1981). Micrognathia, temporomandibular joint changes and dental occlusion in juvenile rheumatoid arthritis of adolescents and adults. Scand J Dent Res.

[B13] Stabrun AE, Larheim TA, Hoyeraal HM (1989). Temporomandibular joint involvement in juvenile rheumatoid arthritis. Clinical diagnostic criteria. Scand J Rheumatol.

[B14] Wallace CA, Ruperto N, Giannini E (2004). Preliminary criteria for clinical remission for select categories of juvenile idiopathic arthritis. J Rheumatol.

[B15] Sidiropoulou-Chatzigianni S, Papadopoulos MA, Kolokithas G (2008). Mandibular condyle lesions in children with juvenile idiopathic arthritis. Cleft Palate Craniofac J.

[B16] Kuseler A, Pedersen TK, Gelineck J, Herlin T (2005). A 2 year followup study of enhanced magnetic resonance imaging and clinical examination of the temporomandibular joint in children with juvenile idiopathic arthritis. J Rheumatol.

[B17] Argyropoulou MI, Margariti PN, Karali A, Astrakas L, Alfandaki S, Kosta P, Siamopoulou A (2008). Temporomandibular joint involvement in juvenile idiopathic arthritis: clinical predictors of magnetic resonance imaging signs. Eur Radiol.

[B18] Weiss PF, Arabshahi B, Johnson A, Bilaniuk LT, Zarnow D, Cahill AM, Feudtner C, Cron RQ (2008). High prevalence of temporomandibular joint arthritis at disease onset in children with juvenile idiopathic arthritis, as detected by magnetic resonance imaging but not by ultrasound. Arthritis Rheum.

[B19] Muller L, Kellenberger CJ, Cannizzaro E, Ettlin D, Schraner T, Bolt IB, Peltomaki T, Saurenmann RK (2009). Early diagnosis of temporomandibular joint involvement in juvenile idiopathic arthritis: a pilot study comparing clinical examination and ultrasound to magnetic resonance imaging. Rheumatology (Oxford).

[B20] Twilt M, Arends LR, Cate RT, van Suijlekom-Smit LW (2007). Incidence of temporomandibular involvement in juvenile idiopathic arthritis. Scand J Rheumatol.

[B21] Rohlin M, Petersson A (1989). Rheumatoid arthritis of the temporomandibular joint: radiologic evaluation based on standard reference films. Oral Surg Oral Med Oral Pathol.

[B22] Hu YS, Schneiderman ED (1995). The temporomandibular joint in juvenile rheumatoid arthritis: I. Computed tomographic findings. Pediatr Dent.

[B23] Cahill AM, Baskin KM, Kaye RD, Arabshahi B, Cron RQ, Dewitt EM, Bilaniuk L, Towbin RB (2007). CT-guided percutaneous steroid injection for management of inflammatory arthropathy of the temporomandibular joint in children. AJR Am J Roentgenol.

[B24] Lamer S, Sebag GH (2000). MRI and ultrasound in children with juvenile chronic arthritis. Eur J Radiol.

[B25] Emshoff R, Brandlmaier I, Bodner G, Rudisch A (2003). Condylar erosion and disc displacement: detection with high-resolution ultrasonography. J Oral Maxillofac Surg.

[B26] Jank S, Emshoff R, Norer B, Missmann M, Nicasi A, Strobl H, Gassner R, Rudisch A, Bodner G (2005). Diagnostic quality of dynamic high-resolution ultrasonography of the TMJ – a pilot study. Int J Oral Maxillofac Surg.

[B27] Jank S, Haase S, Strobl H, Michels H, Hafner R, Missmann M, Bodner G, Mur E, Schroeder D (2007). Sonographic investigation of the temporomandibular joint in patients with juvenile idiopathic arthritis: a pilot study. Arthritis Rheum.

[B28] Twilt M, Schulten AJ, Verschure F, Wisse L, Prahl-Andersen B, van Suijlekom-Smit LW (2008). Long-term followup of temporomandibular joint involvement in juvenile idiopathic arthritis. Arthritis Rheum.

[B29] Scarfe WC, Farman AG, Sukovic P (2006). Clinical applications of cone-beam computed tomography in dental practice. J Can Dent Assoc.

[B30] Huntjens E, Kiss G, Wouters C, Carels C (2008). Condylar asymmetry in children with juvenile idiopathic arthritis assessed by cone-beam computed tomography. Eur J Orthod.

[B31] Kofod T, Norholt SE, Pedersen TK, Jensen J (2005). Unilateral mandibular ramus elongation by intraoral distraction osteogenesis. J Craniofac Surg.

[B32] Ince DO, Ince A, Moore TL (2000). Effect of methotrexate on the temporomandibular joint and facial morphology in juvenile rheumatoid arthritis patients. Am J Orthod Dentofacial Orthop.

[B33] Moen K, Kvalvik AG, Hellem S, Jonsson R, Brun JG (2005). The long-term effect of anti TNF-alpha treatment on temporomandibular joints, oral mucosa, and salivary flow in patients with active rheumatoid arthritis: a pilot study. Oral Surg Oral Med Oral Pathol Oral Radiol Endod.

[B34] Sherry DD, Stein LD, Reed AM, Schanberg LE, Kredich DW (1999). Prevention of leg length discrepancy in young children with pauciarticular juvenile rheumatoid arthritis by treatment with intraarticular steroids. Arthritis Rheum.

[B35] Allen RC, Gross KR, Laxer RM, Malleson PN, Beauchamp RD, Petty RE (1986). Intraarticular triamcinolone hexacetonide in the management of chronic arthritis in children. Arthritis Rheum.

[B36] Aggarwal S, Kumar A (1989). A cortisone-wrecked and bony ankylosed temporomandibular joint. Plast Reconstr Surg.

[B37] Haddad IK (2000). Temporomandibular joint osteoarthrosis. Histopathological study of the effects of intra-articular injection of triamcinolone acetonide. Saudi Med J.

[B38] Kopp S, Carlsson GE, Haraldson T, Wenneberg B (1987). Long-term effect of intra-articular injections of sodium hyaluronate and corticosteroid on temporomandibular joint arthritis. J Oral Maxillofac Surg.

[B39] Martini G, Bacciliero U, Tregnaghi A, Montesco MC, Zulian F (2001). Isolated temporomandibular synovitis as unique presentation of juvenile idiopathic arthritis. J Rheumatol.

[B40] Arabshahi B, Dewitt EM, Cahill AM, Kaye RD, Baskin KM, Towbin RB, Cron RQ (2005). Utility of corticosteroid injection for temporomandibular arthritis in children with juvenile idiopathic arthritis. Arthritis Rheum.

[B41] Schroeder SM, Cannizzaro WE, Kellenberger C, Peltomaki T, Saurenmann RK (2008). Temporomandibular Joint Arthritis in Patients with Juvenile Idiopathic Arthritis: Efficacy of Intraarticular Corticosteroid Injection as Measured by MRI and Clinical Examination. Arthritis Rheum.

[B42] Tzaribachev N, Fritz J, Holzer U, Pereira PL, Horger M, Kuemmerle-Deschner J (2007). JIA – A Silent "Killer" of the Temporo-mandibular Joints. Arthritis Rheum.

[B43] Ringold S, Torgerson TR, Egbert MA, Wallace CA (2008). Intraarticular corticosteroid injections of the temporomandibular joint in juvenile idiopathic arthritis. J Rheumatol.

[B44] Stoustrup P, Kristensen KD, Kuseler A, Gelineck J, Cattaneo PM, Pedersen TK, Herlin T (2008). Reduced mandibular growth in experimental arthritis in the temporomandibular joint treated with intra-articular corticosteroid. Eur J Orthod.

[B45] Kristensen KD, Stoustrup P, Kuseler A, Pedersen TK, Nyengaard JR, Hauge EM, Herlin T (2008). Quantitative histological changes of repeated antigen-induced arthritis in the temporomandibular joints of rabbits treated with intra-articular corticosteroid. J Oral Pathol Med.

[B46] Stoustrup P, Kristensen KD, Kuseler A, Pedersen TK, Gelineck J, Herlin T (2009). Intra-articular vs. systemic administration of etanercept in antigen-induced arthritis in the temporomandibular joint. Part II: mandibular growth. Pediatr Rheumatol Online J.

[B47] Kristensen KD, Stoustrup P, Kuseler A, Pedersen TK, Nyengaard JR, Hauge E, Herlin T (2009). Intra-articular vs. systemic administration of etanercept in antigen-induced arthritis in the temporomandibular point. Part I: histological effects. Pediatr Rheumatol Online J.

